# 

**Published:** 1900-02-17

**Authors:** 


### NOTICE TO CORRESPONDENTS.

All MS., Letters, Books for Review, and other matters intended for the Editor should be addressed The Editor, "The Hospital," The Hospital Building, 28 & 29, Southampton Street, Strand, W.C.

The Editor cannot undertake to return rejected MS., even when accompanied by stamped directed envelope.

**Notice to Advertisers and Subscribers.**

All Advertisements, Orders for Copies of The Hospital, and Business Communications should be addressed to **THE MANAGER (Not to the Editor)**, The Hospital, The Hospital Building, 28 & 29, Southampton Street, Strand, London, W.C.

The Cost of Subscription, post free, is as follows:—Per week, 2½d. per quarter, 2s. 8d.; per half-year, 5s. 3d.; per annum, 10s. 6d. Foreign:—Per annum, 15s. 2d., payable, in all cases, in advance.

**NOTICE.—Vol. XXVI. of "The Hospital" (April, 1899, to September, 1899), in handsome cloth binding, is now on sale at the Office of this Journal, price 7s. 6d. Cases for binding the Numbers contained in this Volume 1s. 6d. Index to Vol. XXVI., 2½d., post free.**

**The Monthly Part for February is now ready. Price 6d., by post 8d.**

### BOOKS RECEIVED.

Methuen and Co.

"A Handbook of Nursing." By M. N. Oxford.

Longmans, Green, and Co.

"A Manual of Pathology." By Joseph Coats, M.D. Fourth Edition. Revised throughout by Lewis R. Sutherland, M.B.

Rebman (Limited).

"The Pasteurisation and Sterilisation of Milk." By Albert E. Bell, F.I.C., F.C.S.

State Charities Aid Association of New York.

Annual Reports of the Association and of the Visiting Committee.

John Wright and Co.

"Golden Rules of Physiology." By J. Walker Hall, M.B., Ch.B., and J. Ackworth Menzies, M.D., C.M. "Golden Rules" Series.

Burlington House, Cambridge.

"Matriculation Directory." January, 1900.

### Scraps and Gleanings.

The proprietors of Mellins Food (Limited) have undertaken to supply gratuitously Mellins Food to all babies who have been rendered fatherless by the deaths of soldiers who have been killed in the war.

New accommodation for the nurses and a new out-patient department are required at the Walsall Hospital. These additions have been estimated at £2,500 to £3,000. In addition to this expense the hospital is in debt, so it is hoped that ample support will be forthcoming.

The receipts during 1899 of the Walsall Hospital Sunday Committee were £434, but those interested desire that this year's totals should amount to £500, which amount would only be a reasonable contribution, they hold, to the Walsall Hospital on the part of the churches.

The Vinolia Company (Limited) are contributing to the Lord Mayor's War Fund one halfpenny on each tablet of Vinolia soap sold. This handsome contribution amounted by the middle of January to about £5,000, and it is hoped by the proprietors that the total amount so sent will reach £25,000.

On the outbreak of the war the committee of Queen Charlotte's Lying-in Hospital undertook to receive into the hospital for their confinements the wives of soldiers and sailors on active service in South Africa. Orders of admission have been issued to 37 women, and 15 others are being attended as out-patients.

The medical staff of the Chelmsford Infirmary and Dispensary are desirous of having the operating theatre refurnished, as the present furniture is out of date. It is estimated that an expenditure of £200 in this direction is necessary. Mr. H. C. Wells has promised £50 of this sum, and Mr. F. Wells promises to give £50 so soon as the secretary has obtained £150 of the required sum.

From the annual report of the Gorleston Cottage Hospital it was seen that in 1899 the expenditure exceeded the income by £57 odd. The income showed an increase of £43 odd over that of the preceding year. The chairman thought that the hospital should receive a portion of the amounts distributed by Sir Savile Crossley from the Somerleyton admission fees and also from the Norfolk and Norwich Festival.

The committee of the Sussex Eye Hospital had to report a considerable falling off in income at the recent annual meeting. Although the Hospital Sunday grant has increased, the contributions from workmen's boxes and the annual subscriptions have both decreased. The decrease in donations is very great, the amount received under this head in 1897 being £625 odd, in 1898 £286 odd, whilst in 1899 it was only £47 17s. 6d. It is this decrease that explains the increasing debt which the hospital s had to incur.

ha

The executive committee of the Cardiff Infirmary have adopted a resolution by which it is provided that the workmen employed in any colliery, shipyard, factory, or workshop, or the members of any trade society or association subscribing £10 to the funds of the infirmary, shall be entitled to nominate one person out of their number as a governor for every £10 subscribed during the year immediately preceding the election of governors. A resolution was also passed to the effect that the societies thus represented should select three members of the executive committee.

At the recent annual meeting of the Glasgow Royal Infirmary it was unanimously resolved to authorise the managers to take such steps as they may consider expedient to obtain the inclusion of women and working men on the Board of Management. The present Board are entirely in favour of this addition, but as the charter of the Royal Infirmary only provides for a Board of Management of ten persons, and as they cannot dispense with any of the present ten, it is hoped that the charter will be amended, with a view to increasing the regulated number of managers, and that next year women will have a seat on the Board.

At the monthly meeting of the committee of Worcester Infirmary a resolution was adopted sanctioning the admission to the benefits of the Infirmary of Worcestershire soldiers and sailors who had served in South Africa, and also their wives and families, on the recommendation of the officials of the Soldiers and Sailors' Families' Association or the Mayor of Worcester's Soldiers and Sailors' Reservists' Fund. The suggestion that a ward should be reserved for soldiers and sailors who might come home wounded was not adopted, as the House Committee thought that the needs of the moment would be better met by the first-named resolution.

A novel scheme of helping charitable funds has just been carried out by Mrs. Pollard, of Torquay, who conceived the idea of turning to profitable account her talent for making confectionery. Accordingly with the assistance of other ladies she manufactured a large quantity of various kinds of sweetmeats, placed them in a shop (lent for the occasion), which was prettily and appropriately decorated, and has been selling them to the Torquay public at the uniform price of 4s. per pound, her saleswomen being Torquay ladies who have interested themselves in the scheme. The first day's sales realised £53, and as the sale is to be continued for one week it is to be expected that Mrs. Pollard will have a handsome sum to hand over to the Syracusa Convalescent Home, which institution has been chosen to benefit by this original and clever plan of raising money.

### Hospitals, &c., Urgently Needing Annual Subscriptions, Donations, and Legacies.

### LONDON HOSPITAL, E.

### The Committee appeal for £40,000 a-year from VOLUNTARY CONTRIBUTIONS.

| | |
|---|---|
| The number of IN-PATIENTS treated in 1897 was | 11,146 |
| " OUT-PATIENTS " " | 161,033 |
| Total number of Patients treated at the Hospital— | 172,179 |

### FUNDS ARE URGENTLY NEEDED.

**Annual Subscribers are entitled to Tickets.**

Thoroughly-Trained Private Nurses to be had immediately on application to the Matron.

HONBLE. SYDNEY HOLLAND, *Chairman.* G. Q. ROBERTS, *House Governor.*

### THE UNIFORM SYSTEM OF ACCOUNTS,

**AUDIT, AND TENDERS, for Hospitals and Institutions, with Certain Checks upon Expenditure; also the Index of Classification.** Compiled by a Committee of Hospital Secretaries, and adopted by a General Meeting of the same, 18th January, 1892. And certain Tender and other Forms for securing Economy, By Sir HENRY BURDETT, K.C.B. Demy 8vo, profusely illustrated with Specimen Tables, Index of Classification, Forms of Tender, &c., cloth extra, 6s.

### ACCOUNT BOOKS FOR INSTITUTIONS,

**designed in accordance with the Uniform System of Accounts for Hospitals and Institutions.** By Sir HENRY BURDETT, K.C.B. Being a complete set of Account Books ruled in accordance with the Uniform System adopted by the Metropolitan Hospital Sunday Fund.

*(Full Description of Books and Prices sent post free on application.)*

London: THE SCIENTIFIC PRESS, Ltd., 28 & 29, Southampton Street, Strand, W.C.

Accouchement Requisites.

Ambulance Cases.

### NOW READY,

A REPRINT OF

### A NURSING HANDBOOK

TOGETHER WITH S. MAW, SON, AND THOMPSON'S

### COMPLETE CATALOGUE OF NURSING REQUISITES.

The above work is now republished at a cost of 1s. per copy. Messrs. S. M., S., and T. have been compelled to make this charge on account of the extreme popularity of the work and the great expense incurred in its production.

***Post Free on receipt of 1s.***

### S. MAW, SON, and THOMPSON,

7 to 12, ALDERSGATE STREET, LONDON, E.C.

